# Effect of traditional teaching and sandwich combination in clinical teaching of granulomatous mastitis

**DOI:** 10.1097/MD.0000000000047762

**Published:** 2026-03-06

**Authors:** Lanying Li, Wen Yang, Haiming Jia

**Affiliations:** a Clinical Medicine School of North Sichuan Medical College, Nanchong City, China; b Breast Surgery Department of the Third Hospital of Mianyang, Sichuan Mental Health Center, Mianyang City, China; c General Surgery Department of Lanzhou Second People’s Hospital, Lanzhou City, China.

**Keywords:** breast surgery, clinical teaching, sandwich teaching, traditional teaching

## Abstract

As an important part of surgery, breast surgery not only involves knowledge of related disciplines, but also involves a variety of disciplines such as psychology, pharmacology and physiology. This study aims to compare the effect of traditional teaching and sandwich joint teaching in the clinical teaching of granulomatous mastitis. A total of 80 breast surgery interns from January 2023 to January 2024 were selected according to different teaching methods, and 40 of them were selected as the control group using the traditional teaching method. The other 40 cases were taken as the experimental group and were taught by the sandwich joint case teaching method. The differences of assessment scores, mini-clinical evaluation exercise scores and stress were compared between the 2 groups after teaching. The scores of test group in theory, practice and comprehensive assessment were higher than those of control group, and the difference was statistically significant (*P* < .05). In terms of mini-clinical evaluation exercise scores, the experimental group was significantly higher than the control group in clinical judgment, medical reading, communication skills, humanistic care, organizational effectiveness, overall performance and teacher satisfaction, with statistical significance (*P* < .05). In terms of Beck stress score, the study and interpersonal stress in the experimental group were significantly higher than those in the control group, and the difference was statistically significant (*P* < .05). The combined sandwich case teaching method can improve students’ grades, problem-solving ability, interaction and communication skills, and reduce learning and interpersonal pressure.

## 1. Introduction

Breast surgery is the next branch of clinical surgery, which mainly focuses on the surgery of breast diseases, including breast anatomy, breast infection, pathology of breast tumor, principles of diagnosis and treatment of breast tumor, diagnosis of breast tumor, various surgical procedures of breast tumor surgery, chemotherapy, radiotherapy, and endocrine therapy. The clinical tasks are arduous.^[1,2]^ With the continuous progress of modern medicine, the diagnosis and treatment results of various gynecological diseases have been effectively improved. The basis for promoting the health outcomes of gynecological patients lies in whether clinicians have professional skills.^[3]^ Clinical learning is a very important link in the learning process of medical students and an important way to make full use of the theoretical knowledge learned in the past,^[4]^ so as to realize the organic combination of textbook theoretical knowledge and practical ability.^[5,6]^ The quality of teaching affects the future work ability of clinical interns to a certain extent, and more scientific research on teaching content and methods has practical significance.^[7]^

Traditional Chinese medical education is passive learning with weak independent learning and lack of independent thinking ability of students.^[8]^ If students are to overcome learning inertia, teachers must improve the teaching design.^[8]^ Sandwich teaching method originated in the 19th century and was proposed by the sandwich course in British universities, which emphasizes the combination of theoretical teaching and practical operation.^[9]^ Later, it was introduced into medical classroom education by the Medical School of Heidelberg University in Germany.^[1]^ Sandwich teaching method is a kind of scientific teaching mode commonly used in clinical intern teaching at present.^[3]^ Sandwich teaching method can effectively cultivate students’ ability to analyze and solve problems through multiple interactive links between teachers and students and students. In the process of interaction, students’ ability of communication and cooperation is also exercised, so as to cultivate their comprehensive ability.^[1,3]^ Studies have found that sandwich teaching has achieved good teaching effect in the teaching application of endocrinology, ophthalmology, pediatric surgery and other disciplines,^[4,5,9]^ but it has rarely been introduced into breast surgery.

Therefore, in order to explore the teaching effect of sandwich teaching method in clinical teaching of breast surgery, this study took 80 breast surgery interns in our hospital from January 2023 to January 2024 as research samples, divided them by random number table method, and selected 40 of them as control group, and taught by traditional teaching method. Another 40 cases were taken as the experimental group, and the teaching method of sandwich joint case was used to teach, and the differences of assessment scores, mini-clinical exercise assessment (Mini-CEX) scores and stress were compared between the 2 groups after teaching.

## 2. Methods

### 2.1. Study design and population

This study was approved by the Ethics Committee of Lanzhou Second People’s Hospital. A total of 80 breast surgery interns from January 2023 to January 2024 were selected as the study samples, and 40 of them were divided by different teaching methods as the control group, including 3 males and 37 females. The mean age was (25.42 ± 3.13) years. Among them, 39 cases had bachelor’s degree and 1 case had a master’s degree. The other 40 cases were experimental group, including 4 males and 36 females; Mean age (25.89 ± 3.01) years; Among them, 38 were undergraduates and 2 were masters. There was no significant difference in gender and age between the 2 groups (*P* > .05), suggesting that this study is comparable and the results obtained are real and reliable. *Inclusion criteria:* All clinical interns in the unit; Teaching in breast surgery or nail and breast surgery; Know the content of this study, be included in the group voluntarily, and sign the consent; With normal expression ability. *Exclusion criteria:* Withdrawal of researchers; Cognitive dysfunction; Poor independent learning ability. All participants were interns undergoing the same stage of standardized clinical rotation in breast surgery, and no cross-major or cross-grade recruitment was involved.

### 2.2. Teaching methods

In the control group, traditional teaching method was implemented, one-to-many clinical teaching was carried out, conventional breast surgery teaching training was carried out, assessment was carried out after the teaching period, and questionnaires were collected. While, in experimental group, on the basis of traditional teaching, combined sandwich teaching method, see the following: Opening: Introduce the learning content, randomly group the interns to sit, and lead the teacher to “non-lactating women, will not mastitis occur?” For the topic, introduce the teaching content; Around the topic, the following questions are put forward to guide students’ thinking and learning: “Will mastitis not occur in non-lactating women?,” “Why say it is ‘duplicity’ immortal cancer?,” “How does granulomatous mastitis happen?,” or “How is it treated?”; Intragroup discussion: Each intern participates in the intragroup discussion according to the treatment plan in hand, boldly expresses academic views and seeks common ground while reserving differences. This stage can effectively improve the intern’s independent thinking and adaptability. Finally, the representative of the group will read out the final treatment plan in the group and accept questions from other groups; Teacher’s summary: The teacher will make a summary according to the content reported by the students, and encourage the students to interact with each other during the summary process, help the students reflect on the process of independent learning, check the gaps and make up for the weaknesses; Feedback: Students will give comments and feedback on the teaching content from the aspects of how to prepare before class, learning ability and learning harvest; Answer questions: the teacher answers all kinds of doubts raised by the students and assigns homework after class. Students are asked to understand the various methods of treatment for patients with granulomatous mastitis by reviewing the literature. No data and shedding numbers were available in this study.

### 2.3. Examination results

After the end of the teaching, each group of interns will be assessed at the end of the semester, which is divided into theoretical, practical and comprehensive assessment, with a full score of 100 points. The higher the score, the better the quality of the teaching.

### 2.4. Mini-clinical exercise assessment

After the teaching, Mini-CEX^[10]^ was applied to nursing students, including 7 dimensions of clinical judgment, medical reading, communication skills, humanistic care, organizational effectiveness, overall performance, and teacher satisfaction. Each dimension adopted a 9-point scale, and scores ≥ 7 indicated excellent.

### 2.5. Stress

The Beck–Srivastava Stress Inventory^[6]^ was used to evaluate the 4 aspects of learning pressure, economic pressure, interpersonal pressure and practical pressure of the 2 groups of nurses, with a total of 21 items. The 3-level scoring method was adopted, with a total score of 63 points. The higher the score, the greater the pressure.

### 2.6. Statistical methods

Statistical Package for the Social Sciences 17.0 statistical software was used. Measurement data were represented by mean ± SD, *t* test was used, counting data were represented by (%), and *χ*^2^ test was used. *P* < .05 was considered statistically significant.

## 3. Results

### 3.1. Comparison of theoretical, practical, and comprehensive assessment scores

Both the theoretical assessment score and the practical assessment score of the experimental group were higher than those of the control group, and the comprehensive assessment score of the experimental group was also higher than that of the control group, with statistical significance (*P* < .05) (Table 1).

**Table 1:** Comparison of theoretical, practical and comprehensive assessment scores between the 2 groups.

Group	Theoretical examination	Practice assessment	Comprehensive evaluation
Experimental group	91.90 ± 3.15	92.68 ± 2.03	91.53 ± 2.33
Control group	89.13 ± 1.34	88.90 ± 1.41	90.43 ± 2.04
*t*	6.932	9.657	2.247
*P*	.000	.000	.027

### 3.2. Mini-CEX score

After teaching, the scores of Mini-CEX in the test group were higher than those in the control group, and the difference was statistically significant (*P* < .05). Table 2 shows the scores of Mini-CEX in the 2 groups.

**Table 2:** Comparison of Mini-CEX scores between 2 groups of students.

Group	Clinical judgment	Medical reading	Communication skills	Humanistic care	Organizational effectiveness	Overall performance	Teacher satisfaction
Experimental group	8.00 ± 0.68	7.48 ± 0.60	7.30 ± 0.65	7.43 ± 0.59	7.70 ± 0.85	7.58 ± 0.68	8.10 ± 0.63
Control group	6.13 ± 0.94	6.88 ± 0.91	6.50 ± 0.96	6.90 ± 0.59	6.65 ± 0.74	6.53 ± 0.85	6.78 ± 0.58
*t*	10.233	3.481	4.365	3.963	5.895	6.131	9.790
*P*	.000	.001	.000	.000	.000	.000	0.000

### 3.3. Beck stress

After teaching, the score and practice stress in Beck stress score of experimental group were lower than those of control group, and the difference was statistically significant (*P* < .05), while there was no statistically significant difference in economic pressure and interpersonal pressure between the 2 groups (*P* > .05) (Table 3).

**Table 3:** Comparison of Beck stress scores between 2 groups of students.

Group	Student stress	Economic pressure	Interpersonal stress	Practical pressure
Control group	3.43 ± 0.68	3.28 ± 0.75	3.43 ± 0.68	3.40 ± 0.74
Experimental group	2.98 ± 0.78	3.05 ± 0.81	3.10 ± 0.78	3.33 ± 0.73
*t*	2.784	1.284	1.996	0.455
*P*	.007	.203	.049	.650

## 4. Discussion

Sandwich teaching method cultivates students’ ability to combine theory and practice through the alternations of theoretical learning and work practice, and emphasizes that “students are the main body of the classroom,” thus establishing a teacher-guided and student-led classroom teaching system.^[11,12]^ Compared with the conventional teaching mode, the “sandwich” teaching mode in the prescribed teaching time is more likely to improve students’ independent learning and research ability. The “sandwich” teaching model is applied to clinical teaching to better apply the theory. Organic combination with clinical practice, and realize the multi-angle analysis of breast surgery theoretical knowledge, the classroom teaching design under this model is interlinked and interrelated, and can improve the rigor of classroom theoretical teaching and teaching effect.^[13–15]^ The results of this study show that the combined sandwich case teaching method can improve students’ achievement.^[16,17]^ In addition, the sandwich teaching method effectively cultivates students’ ability to analyze and solve problems through multiple interactive links between teachers and students and students. In the process of group cooperation, students help each other, make full use of class time, and learn how to reach a consensus in the process of discussion.^[18–20]^

Sandwich teaching method builds the teaching process on the basis of students’ active exploration and practice, allowing students to observe, explore and think about problems from different angles, and integrate the knowledge learned in the process of repeated discussions, so that students can truly understand and learn to apply classroom knowledge.^[21–24]^ The results of this study showed that the scores of clinical judgments, medical reading, communication skills, humanistic care, organizational effectiveness, overall performance and teacher satisfaction of interns under the “sandwich” teaching mode were higher than those of traditional teaching.

Clinical practice for medical students is an important period in which theory is combined with practice, and it is also the most critical stage in cultivating high-quality and high-quality medical talents. Due to the lack of professional theory and skills and the relative lack of communication ability, various pressures such as anxiety, fear, tension, impatience, and inferiority will occur when facing various problems.^[25,26]^ In the face of the above phenomena, the “sandwich” teaching mode can effectively reduce the psychological pressure of interns, help to release psychological pressure, increase clinical learning self-confidence, and improve learning efficiency.^[27–29]^ The results of this study showed that the scores of learning stress and interpersonal stress in the observation group were lower than those in the control group, indicating that the “sandwich” teaching model can effectively reduce the learning and practice stress of new nurses. But the sandwich teaching method also has some limitations. For example, the teaching method has higher requirements on teachers and requires a larger learning space for discussion. Besides, students are used to the infusing teaching mode in class. In sandwich teaching, they need to take the initiative to find answers, and the adaptation process is slow, so the teaching method takes slightly longer time than the traditional method.

The applicability of the sandwich teaching method may vary across different grades and medical majors. Junior students may benefit more from structured guidance, while senior students may gain greater advantages from independent discussion-based learning. This study has several limitations, including its single-center design, relatively small sample size, and lack of long-term follow-up outcomes. Future multi-center studies with larger samples are warranted.

## 5. Conclusion

In summary, the combined sandwich case teaching method can improve students’ grades, problem-solving skills, interaction and communication skills, and reduce academic and interpersonal stress. Therefore, sandwich teaching reform is an important exploration of the reform of medical teaching mode under the new situation, and has achieved good teaching effect, and has made a beneficial attempt to deepen the teaching of mammary internal medicine in the future.

## Acknowledgments

The authors are grateful to all participants in the present study.

## Author contributions

**Conceptualization:** Lanying Li, Wen Yang, Haiming Jia.

**Data curation:** Lanying Li, Wen Yang, Haiming Jia.

**Formal analysis:** Lanying Li, Wen Yang, Haiming Jia.

**Funding acquisition:** Lanying Li, Wen Yang, Haiming Jia.

**Investigation:** Lanying Li, Wen Yang, Haiming Jia.

**Writing – original draft:** Haiming Jia.

**Writing – review & editing:** Haiming Jia.
